# The 'Zero Tolerance Village Alliance': A promising intervention for addressing sexual and gender-based violence in rural communities

**DOI:** 10.1186/1753-6561-9-S4-A4

**Published:** 2015-07-07

**Authors:** Fiona Nicholson, Craig Carty

**Affiliations:** 1 Thohoyandou Victim Empowerment Programme, P. O. Box 754, Sibasa 0970, Thohoyandou, Limpopo, South Africa; 2 The Relevance Network, 29 Pentrich Road, Suite 104, Johannesburg 2195, Gauteng, South Africa

## Background

Worldwide, few countries have higher rates of sexual and gender-based violence (SGBV) than South Africa [1]. From 2013 to 2014 alone, nearly 50,000 rape incidents were reported to the police in the country [2]. This predicament affects the young and the old: the majority of sexual violence survivors presenting at Thuthuzela Care Centers are less than 18 years of age (60%), and 40% of all rape survivors are pre-teens under the age of 12 [1]. Nonetheless, annual reporting rates of rape in the country are comparatively low, with only 1 in 9 rapes reported each year [3]. Furthermore, the issue of sexual violence in South Africa is compounded by the linkages between such violence and HIV seropositivity [4]. Given these realities, this study sought to assess the effects of an intervention geared toward positively influencing negative community norms related to SGBV.

## Materials and methods

### Study design

The study used a pre- and post-intervention design with a comparison group, covering villages in the Thohoyandou region of Limpopo Province in South Africa. Two villages (Lunungwi and Tshiombo) served as the intervention sites, while one village (Mangondi) served as the comparison site.

### Intervention

The intervention was implemented by the Thohoyandou Victim Empowerment Programme in rural South Africa over a 9-month period from May 2011 to January 2012. Referred to as the 'Zero Tolerance Village Alliance' (ZTVA), the intervention was premised on the notion that changing SGBV norms would require the involvement of all community members (particularly men and women), and that, ultimately, a village would have to take ownership of the issue of SGBV, understand the need to ensure a 'zero-tolerance' environment against SGBV, and demonstrate motivation to have their community branded in this way. Addressing these issues would be likely to reduce levels of SGBV in communities, lessen stigma around admitting personal experiences of SGBV, and increase awareness of where to obtain care for SGBV. Being branded as a 'zero-tolerance' village might also help ensure the sustainability of any gains made as a result of the intervention.

The ZTVA model comprised several different components geared toward mobilizing communities to come together in a united effort against SGBV. These components included:

• a series of community dialogues to introduce and promote ownership of the ZTVA model

• the appointment of a Stakeholders Forum representative of community structures and agencies (e.g., traditional authority, churches, schools, businesses, and civil society agencies), and responsible for facilitating and monitoring intervention activities

• the development of a Memorandum of Agreement (MoA) outlining the criteria for a village's induction into the Zero Tolerance Village Alliance

• the development of community maps to highlight opportunities for partnerships to enhance SGBV service provision, and to disseminate SGBV messages

• training of Stakeholder Forum members on SGBV good governance procedures and policies, rights and responsibilities, and accountability monitoring (this training includes a 'Training of Trainers' module to ensure cascading of training to other community groups)

• promoting the attainment of ZTVA criteria, including (but not limited to):

• participation of at least 1,250 adults and youth per village in a series of five workshops or dialogues, covering SGBV issues and accountability monitoring

• adherence of government service providers to their relevant and respective mandates (e.g., police trained in victim empowerment, clinics displaying Victim's Charter and providing male and female condoms)

• existence of: a short-term, community-run safe house for victims of domestic violence; and a functioning support group for people living with HIV and orphans and vulnerable children

Once the village concerned meets all the induction criteria listed above, the final aspect of the intervention involves a public ceremony during which men of the village are invited to make a public pledge to proactively address the eradication of SGBV in their village. Pledgers are asked to sign a 'Roll of Honor,' and given a 'Badge of Honor' to identify them as men who have taken a zero-tolerance stance. Women are given a 'Badge of Courage' to promote their agency to report abuse. The ceremony culminates in the unveiling of a large billboard in the inducted village, declaring its zero-tolerance status.

### Data collection and analysis

Household survey data were collected from women and men via structured questionnaires before and after the intervention in all study sites. Descriptive statistics on each variable at baseline and endline constituted the principal procedure for data analysis, in addition to comparison of baseline and endline information from intervention and comparison villages. The data were analyzed using the Minitab Release 14.20 statistical software package.

## Results

A total of 1,134 and 1,180 women and men participated in the baseline and endline household surveys, respectively, from all 3 villages combined. Selected results presented in this paper center on self-reported experiences of SGBV, knowledge of where to access post-rape care services, and gender beliefs about sexual refusal in intimate partnerships.

### Self-reported experiences of SGBV

**Figure 1:**
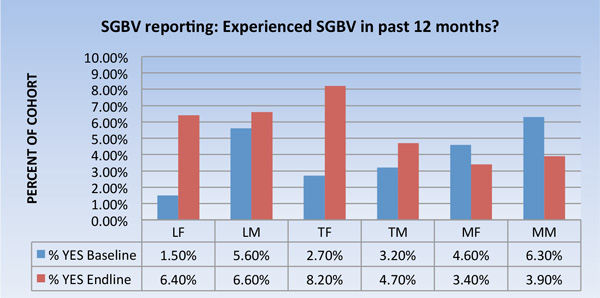
**SGBV Self-Reporting Data.** LF=Lunungwi females; LM=Lunungwi males; TF= Tshiombo females; TM= Tshiombo males; MF=Mangondi females; MM=Mangondi males

Self-reported experiences of SGBV increased in the two ZTVA intervention villages and decreased in the comparison village (Figure 1). The observed changes in the intervention and comparison sites were not statistically significant, however.

### Knowledge of where to access post-rape care

**Figure 2:**
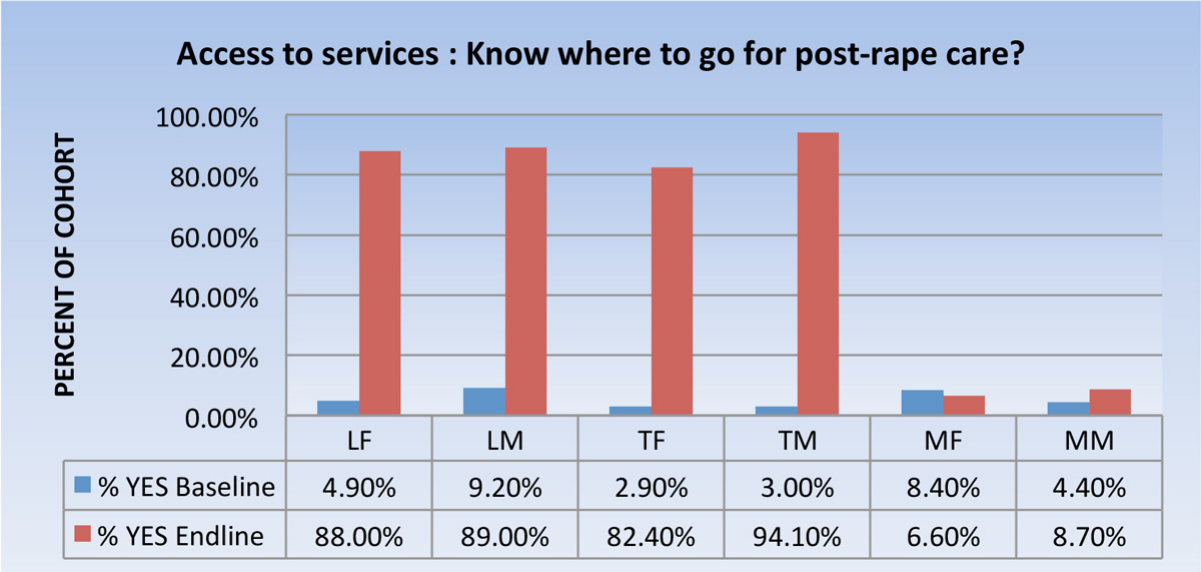
**Access to Services** LF=Lunungwi females; LM=Lunungwi males; TF= Tshiombo females; TM= Tshiombo males; MF=Mangondi females; MM=Mangondi males

A crucial factor in reporting and seeking care in cases of SGBV hinges upon the knowledge of where to seek such care. Knowledge of where to obtain this kind of care increased in the ZTVA villages (Figure 2). This increase was statistically significant in both intervention sites. In the comparison village, on the other hand, a slight decline in knowledge was observed among women, while a slight, non-statistically significant increase was observed among men.

### Gender beliefs

By endline, there was a statistically significant increase in the proportion of female and male respondents in Lunungwi (one of the ZTVA intervention villages) who strongly agreed with the notion that women had the agency to refuse to have sex with their partner. Slight increases in this regard were also observed in Tshiombo (the second ZTVA village), but these were not statistically significant.

In contrast, the comparison village (Mangondi) saw a reduction in the proportion of men who believed a woman has the power to decline sexual advances from her partner. There were also reductions in the comparison village in the proportion of women and men alike who agreed (as opposed to 'strongly agreed') with this statement. None of the changes observed in the comparison village were statistically significant.

**Figure 3:**
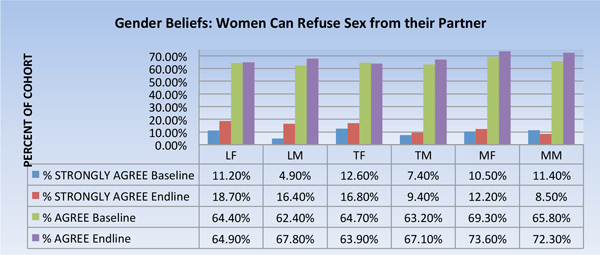
**Gender Beliefs.** LF=Lunungwi females; LM=Lunungwi males; TF= Tshiombo females; TM= Tshiombo males; MF=Mangondi females; MM=Mangondi males

## Conclusions

The study findings suggest that the 'Zero Tolerance Village Alliance' model holds promise for changing community norms around SGBV, including potentially countering stigma related to reporting SGBV experiences, increasing awareness of where to access SGBV care, and promoting gender equality. As community-based interventions often require a lengthy period of implementation before significant improvements are observed [5], it is plausible that the ZTVA implementation period of 9 months was too brief for more of such improvements to be registered in the area of SGBV. Nonetheless, as few SGBV-focused, community-wide interventions exist in the sub-Saharan African region, efforts should be made to strengthen the ZTVA model and to implement it over a longer period of time in order to understand its full potential. Doing so is particularly important as the Thohoyandou Victim Empowerment Programme has received requests from several village elders in Limpopo Province to be inducted into the Zero Tolerance Village Alliance. Furthermore, since the completion of this study, the organization has received funding to replicate the ZTVA approach in 4 additional villages, and to adapt the ZTVA model to the needs of children in school, and of refugee populations. The organization has also been contracted by donors (including the South African government) and to train a total of 10 community-based organizations in South Africa in the implementation of the ZTVA methodology.
